# Dual Laser Beam Processing of Semiconducting Thin Films by Excited State Absorption

**DOI:** 10.3390/ma14051256

**Published:** 2021-03-06

**Authors:** Christoph Wenisch, Sebastian Engel, Stephan Gräf, Frank A. Müller

**Affiliations:** Otto Schott Institute of Materials Research (OSIM), Friedrich Schiller University Jena, Löbdergraben 32, 07743 Jena, Germany; christoph.wenisch@uni-jena.de (C.W.); sebastian.engel@uni-jena.de (S.E.); frank.mueller@uni-jena.de (F.A.M.)

**Keywords:** dual laser beam processing, excited state absorption, semiconducting thin films, multi-beam micromachining, nanosecond laser, stimulated emission depletion

## Abstract

We present a unique dual laser beam processing approach based on excited state absorption by structuring 200 nm thin zinc oxide films sputtered on fused silica substrates. The combination of two pulsed nanosecond-laser beams with different photon energies—one below and one above the zinc oxide band gap energy—allows for a precise, efficient, and homogeneous ablation of the films without substrate damage. Based on structuring experiments in dependence on laser wavelength, pulse fluence, and pulse delay of both laser beams, a detailed concept of energy transfer and excitation processes during irradiation was developed. It provides a comprehensive understanding of the thermal and electronic processes during ablation. To quantify the efficiency improvements of the dual-beam process compared to single-beam ablation, a simple efficiency model was developed.

## 1. Introduction

In 1994, Hell et al. theoretically described the stimulated emission depletion (STED) microscopy, which was experimentally demonstrated a few years later and today enables to resolve structures smaller than 10 nm [1,2,3]. Here, a Gaussian laser beam excites carriers, which are subsequently depleted back to their ground state by a second donut-shaped STED beam with a specific material-dependent wavelength. Although the focusability of both laser beams is still limited by diffraction, the resulting fluorescent volume is significantly decreased to the zero-intensity center of the STED beam [4].

This approach is now also used for structuring different photopolymers with feature sizes in the low double-digit nm range [5]. In this case, polymerization in the outer, high-intensive region of the donut-shaped beam is suppressed by stimulated depletion of the electrons required for polymerization or by activation of inhibitors. Unfortunately, the technique is limited to certain photopolymers and specific light sources. As an alternative, the structures can be transferred to other classes of materials by replica casting. However, direct sub-diffraction structuring of semiconductors and dielectrics using the STED-technique would improve flexibility, reduce process complexity, and lower overall manufacturing costs [6,7].

In this context, an approach proposed by Engel et al. [8] is very promising for optically active materials like direct semiconductors. Briefly, comparable to STED microscopy, a diffraction limited Gaussian laser beam with sufficient photon energy E_P_ is used to excite electrons into the conduction band (CB). A second temporally and spatially aligned donut-shaped beam with suitable E_P_ in the range of the optical band gap energy E_BG_ depletes these excited states and transfers the electrons back to the valence band (VB) by stimulated emission. The depletion only occurs in the high-intensive ring-shaped region of the beam, whereas the excited electrons in the zero-intensity center keep unaffected. This excited central region can subsequently absorb a third Gaussian laser beam by excited state absorption (ESA) at a wavelength otherwise transmitted in non-excited regions. Based on several processes such as intra-band absorption, avalanche effects, or coulomb explosion, the excess energy provided by this ESA-based process step is potentially suitable for sub-diffraction structuring, although the single beams are subject to diffraction limited focusing.

**Figure 1:**
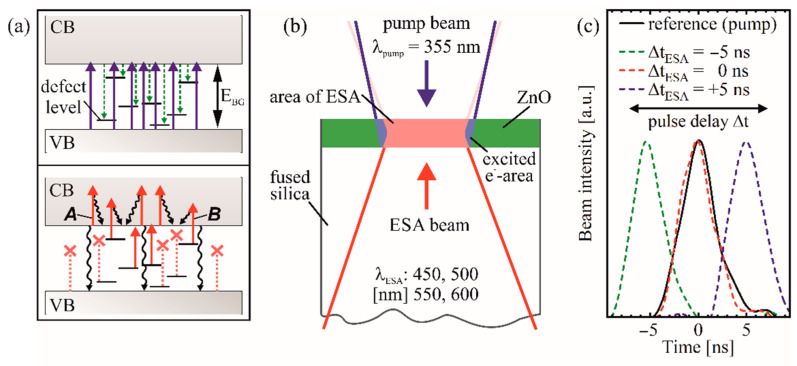
(**a**) Schematic illustration of the theoretical excited state absorption (ESA)-based ablation process with excitation (top) and a subsequent ESA (bottom), indicated by solid arrows. The electrons, excited prior by the pump beam with E_P_ > E_BG_, rapidly relax radiative or non-radiative (dashed arrows) and partially migrate to defect levels located within the band gap. The subsequent ESA beams with E_P_ < E_BG_ are absorbed by either intra-band ***A*** or defect level → conduction band (CB) ***B*** transitions, both enabled only by the prior pump excitation. Waved and dashed arrows indicate non-radiative and forbidden transitions, respectively. (**b**) Sample cross section of 1 mm thick fused silica substrate coated with a 200 nm thin ZnO layer. The beam directions are indicated by arrows. (**c**) Illustration of selected pulse delays Δt = −5, 0, +5 ns (dashed lines) and their temporal overlap with the pump pulse (solid line).

Based on previous studies [9], the focus of the present work is on the ESA-based ablation as an important element of the proposed sub diffraction direct laser writing technique (Figure 1a) [8]. Several studies already implemented an ESA-based microfabrication by a hybrid laser process of wide bandgap semiconductors such as fused silica, sapphire, or quartz [7,10,11,12,13]. The non-thermal ablation by the excitation of electrons above the vacuum energy improves ablation quality and decreases debris formation on the surface. For these dual-beam processes, Excimer and Raman lasers with wavelengths in the UV-range and (ultra-)short pulse lasers are most commonly used to increase absorption through photodissociation and changes in transient absorption [7]. This process is also applicable to lower bandgap semiconductors like gallium nitride, lithium niobate, and silicon carbide [13,14,15,16,17]. In this case, the single laser beams already exceed E_BG_, but the excitation beyond vacuum energy for improved non-thermal ablation is only achieved by the dual-beam setup. Similarly, an increased absorption of a second, otherwise transmitted laser beam with E_P_ < E_BG_, without exceeding the vacuum energy enables energy and cost efficient processing as already demonstrated for silicon and silicon carbide [18,19]. The key factor here is that commercially available, compact, and less expansive lasers in the visible and infrared spectrum can be used [18].

Based on this dual-beam ablation technique, Zinc(II) oxide (ZnO) as a direct bandgap semiconductor was used. Especially as thin films on an low absorbing substrate like fused silica, ZnO is commonly used in state-of-the-art applications like optoelectronics, metamaterials, or photonics, and is therefore a promising candidate for the ESA- and potential STED (in combination with ESA)-based ablation [20,21]. Thus, we focused on the selective, high quality thin film ablation at optimized processing parameters and cost efficiency. To analyze the suitability of the dual-beam setup for thin film structuring, the ablation process is studied with respect to the laser peak fluences, temporal delay, and wavelengths of the respective beams.

## 2. Materials and Methods

### 2.1. Sample Preparation and Characterization

Fused silica substrates (Infrasil 301, Heraeus, Hanau, Germany) with a thickness of 1 mm were coated by RF-magnetron sputtering of 200 nm thin ZnO layers using a sputtering time of 37 min at a pressure of 0.295 Pa, a gas flow (argon with 2% oxygen) of 6 cm³/min, and a power of 150 W. The coated and uncoated samples were optically characterized by UV/VIS transmittance spectroscopy (MCS/100-3, J&M Analytik, Essingen, Germany).

The photoluminescence emission spectra of the coated samples were measured at room temperature with a HeCd laser (IK3202R-D, Kimmon Koha, Tokyo, Japan) at a laser wavelength λ = 325 nm and a continuous wave power of 17 mW. The fluorescence was detected at an angle of 45° by an optical spectrometer (Maya2000 Pro, Ocean Insight, Rostock, Germany). A 350 nm longpass filter (XUL0350, Asahi Spectra Co., Tokyo, Japan) was utilized in front of the detector to restrict the detection range above E_BG_, where only weak emission is expected [22].

### 2.2. Laser Processing Setup

The dual-beam setup consists of two Nd:YAG lasers (SLI-10, Amplitude Systems) with a fundamental wavelength λ = 1064 nm. The third harmonic of one of them was used as pump beam with λ_pump_ = 355 nm and a pulse duration of τ_pump_ = 4.7 ns. The second Nd:YAG laser equipped with an additional optical parametric oscillator provides selected ESA beam wavelengths λ_ESA_ = 450, 500, 550, and 600 nm with τ_ESA_ = 4.8 ns. The beams were collinearly focused on the sample surface from opposite sides. Consequently, the ESA beams must pass the fused silica substrate (Figure 1b). For synchronization and temporal peak-to-peak delay, adjustments of both beams at the sample surface, a digital delay generator (DG535, Stanford Research Systems) and a silicon photodetector (ET-2000, EOT) were used. A digital oscilloscope (XDS3302 Plus, OWON Technology Inc., Zhangzhou, China) with a rise time < 1.2 ns monitored the temporal pulse shape (Figure 1c).

The ablation process was investigated using single laser pulses of the respective pump and ESA beams in order to exclude accumulation and incubation effects that would lead to an altered absorption. These include steady sample heating, contamination of the surface by resolidified ZnO, or the formation of deep level defects and color centers [11,23,24]. To ensure optimum laser operation with highest output stability, the laser was operated at its standard repetition rate of 10 Hz during the measurements. The separate pulses were then selected by mechanical shutters, synchronized with the delay generator already used for laser operations. Furthermore, the lasers were operated at their optimum output energy E_pump_ = 160 mJ and E_ESA_ = 27–29 mJ and attenuated by neutral density filters (FW2AND and NDC-100C-4M, Thorlabs Inc., Newton, MA, USA). To further reduce the impact of pulse energy fluctuations and sample inhomogeneities, the results of 5 independent ablation spots fabricated with identical processing parameters were averaged for all performed single- and dual-beam ablation measurements.

### 2.3. Generation and Analysis of Ablation Spots

The ablation behavior of the ZnO layer for the single laser beams was characterized by optical microscopy (VH-Z100, Keyence, Osaka, Japan) and white light interferometry (CCI HD, Ametek Inc., Berwyn, IL, USA) as a function of their respective pump-pulse energy E_pump_ ≤ 13 µJ and ESA-pulse energy E_ESA_. The latter was labeled according to the utilized ESA-wavelengths λ_ESA_ as E_450_ ≤ 19 µJ, E_500_ ≤ 12 µJ, E_550_ ≤ 25 µJ, E_600_ ≤ 31 µJ. The single-beam ablation threshold energy E_th_ and the beam diameter 2ω_f_ (1/e^2^-diameter) were determined using the method proposed by Liu [25]. For this purpose, the squared ablation diameters D^2^ were plotted in semilog plot versus the pulse energy E. According to

(1) D2 = 2ωf · ln(EEth),

the intersection of the linear fit at D^2^ = 0 and its slope determines E_th_ and 2ω_f_, respectively. Using these parameters, the laser peak fluence at the ablation threshold was calculated by F_th_ = 2E_th_/πω_f_^2^. Hereinafter, all fluence values refer to laser peak fluences.

For fluences below F_th_ in order to prevent single-beam ablation, ESA-based laser processing with a combination of pump and ESA beam was analyzed. The impact of the different parameters for optimal ablation quality and efficiency was characterized by varying the ESA beam fluence F_ESA_ (F_450_ ≤ 1.6 J/cm², F_500_ ≤ 4.5 J/cm², F_550_ ≤ 5.7 J/cm², F_600_ ≤ 5.3 J/cm²) below their individual F_th_ at a constant pulse delay of Δt = 5 ns and several pump beam fluences at F_pump_ = 0.2 J/cm², 0.1 J/cm², and 0.02 J/cm². Using the highest pump beam fluence F_pump_ = 0.2 J/cm^2^ and the same values of F_ESA_, Δt was varied from –10 to 500 ns to investigate the influence of the pulse delay. Here, a negative temporal delay (Δt < 0) implies that the ESA beam precedes the pump pulse (Figure 1c).

## 3. Results and Discussion

### 3.1. Optical Characterization of the ZnO Thin Film

**Figure 2:**
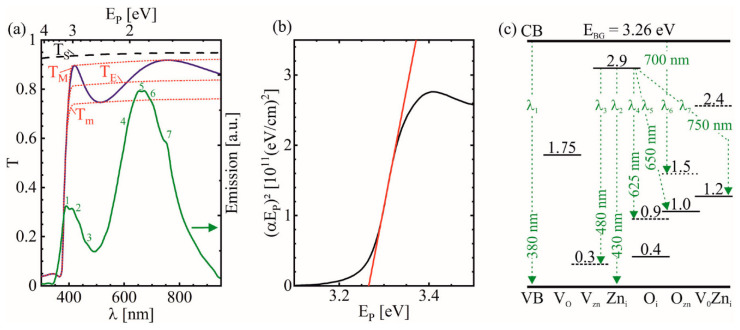
(**a**) UV/VIS transmittance spectra of 1 mm thin uncoated fused silica substrate (**⁃ ⁃ ⁃**) and fused silica coated with a 200 nm thin ZnO-layer (▬). The dotted lines labeled T_M_, T_m_, and T_E_ are the fits of the upper and lower envelope function as well as their geometric mean, respectively [26]. In the emission spectrum of the ZnO film (▬), λ = 325 nm was used for excitation. Selected emission peaks assigned to specific transitions are numbered. (**b**) Tauc plot [28] of T_ZnO_ (▬). The linear fit (▬) at the straight portion of the band edge allows to determine E_BG_ by the intersection at αE_P_ = 0. (**c**) Schematic of the location of intrinsic point defect levels within the band gap by full-potential linear Muffin-tin orbital method calculations according to Xu et al. [32]. The numbers on the lines indicate ΔE_P_ of the defect states compared to the energy of the valence band (VB)-maximum with filled (▬), partially filled (---), and empty (⁃ ⁃ ⁃) states. The positions of the energy levels are true to scale with respect to E_BG_ = 3.26 eV. Selected transitions are labelled with dashed arrows and a number corresponding to the emission wavelengths in (**a**).

The UV/VIS transmittance spectrum of the uncoated fused silica substrate T_S_ (Figure 2a) shows no absorption for the applied λ and thus no interaction with the radiation. The deviation to T = 1 is caused by reflectance losses at the interfaces [26]. The transmittance of the 200 nm thin ZnO layer on the substrate T_ZnO_ shows a single steep absorption edge at λ ≈ 380 nm with a direct optical band gap at E_BG_ = 3.26 eV [20,27], determined by a Tauc-plot [28]:

αE_P_ = G · (E_P_ − E_BG_)^R^, (2)

where G is a constant that depends on the transition probability. The parameter R is determined by the optical transmittance type and is R = 0.5 for directly allowed optical transmissions [29,30]. Plotting αE_P_ over E_P_ and using a linear fit of the straight portion of the absorption edge, the intersection at αE_P_ = 0 determines E_BG_ (Figure 2b). The results demonstrate optimal conditions for a strong fundamental single photon absorption of the pump beam (λ_pump_ = 355 nm). The optical penetration depth is determined by d_p_ = 1/α with the absorption coefficient α = ln(T^−1^)/d_L_. d_p_ = 62 nm and corresponds by definition to the depth at which the beam intensity has dropped to 1/e. This value is smaller than the layer thickness d_L_ and thus almost all of the pulse energy is used to promote electrons to the CB, which are available for the subsequent ESA-based ablation process.

By analyzing the sinusoidal interference fringes in the weak and medium absorbing region of the T_ZnO_-spectrum by the envelope method of Swanepoel [26], the interference free transmittance T_E_ was derived. Here, the T_ZnO_-maxima and minima were used to fit an upper T_M_ and lower T_m_ envelope function, respectively. Both envelopes were computer-generated using the program OriginPro 2020b (Origin-Lab Corp.) [30]. At shorter wavelengths in the strong absorbing region above E_BG_, the envelope functions converge to a single curve, merging with the measured values of T_ZnO_. The interference free absorption coefficient was calculated from the determined T_E_ values (Table 1) [31].

Furthermore, the amplitude and oscillation of the interference fringes contain information about the ZnO-layer thickness d_L_ and the refraction index n, respectively. The refraction index of the substrate was derived from the transmittance of the uncoated substrate. The interference free refraction index in the weak absorbing region was calculated using the envelope functions as demonstrated by Swanepoel [26]. By using λ and n of adjacent fringe maxima or minima and the order number, d_L_ was calculated to be 203 nm. The measured and calculated parameters for the utilized λ are listed in Table 1 [26,31].

Below the band gap energy E_BG_, n and α steadily increase and the small variation of λ below E_BG_ indicates the comparably weak absorption of the ESA wavelengths without additional pump excitation. The difference between T_E_ and T_S_ is caused primarily by the deviating n, as the upper envelope function that intersects the fringe maxima almost coincides with T_S_ [26]. With increasing absorption at E_BG_, α increases rapidly.

The sinusoidal interference fringes in the spectrum are caused by reflection losses due to interference effects of the radiation between the air–film and film–substrate interfaces [33]. This indicates homogeneous, smooth, and low scattering surfaces [34]. Thus, only at sufficiently high beam fluences, an independent ESA beam ablation is expected by either a multi-photon absorption or processes including various intrinsic point defects. The latter include deep level defects inside the ZnO layer or shallow donors that are located typically at the surface [20,22,35,36]. As illustrated in Figure 2c, the location of the intrinsic defect levels were calculated by Xu et al. with a full-potential linear Muffin-tin orbital method [32]. These include vacant zinc (V_Zn_), vacant oxygen (V_O_), interstitial zinc (Zn_i_), interstitial oxygen (O_i_), substituted oxygen at zinc positions (O_Zn_), and complexes of V_0_ and Zn_i_ (V_0_Zn_i_).

The emission spectrum in Figure 2a shows a weaker emission in the UV-range from 360–500 nm (close to E_BG_), compared to λ = 550–800 nm. The sharp band emission at λ_1_ = 380–390 nm overlaps with the band edge and is caused by recombination of photo-induced charge carriers through exciton–exciton collision processes (Figure 2c) [21,22,37]. The specific emissions at λ_2_ = 430 nm and λ_3_ = 480 nm are attributed to Zn_i_ → VB and Zn_i_ → V_Zn_ transitions, respectively [22]. Their weak emission indicates low roughness and homogeneous surface quality, in line with the findings in the T_ZnO_ spectrum as these transitions occur primarily at the surface [22]. The strongest emission in the visible region corresponds to deep level defects in the bulk of the layer [22,32,36]. Here, the broad emission is comprised of several transitions at λ_4_ = 625 nm, λ_5_ = 650 nm, λ_6_ = 700 nm, and λ_7_ = 750 nm, induced by Zn_i_ → O_i_, Zn_i_ → O_Zn_, CB → O_Zn_, and Zn_i_ → V_0_Zn_i_ [22].

### 3.2. Single-Beam Ablation

**Figure 3:**
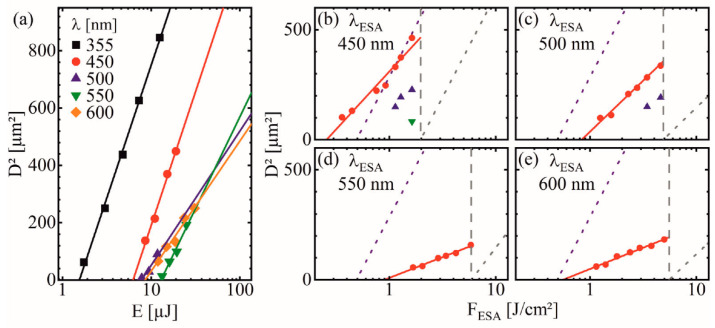
(**a**) Semilog plots of D^2^ over E with the corresponding linear fits, to determine the ablation parameters E_th_, 2ω_f_, and F_th_ of single-beam absorption by the method of Liu [25]. The exact values at the utilized λ are listed in Table 1. (**b**–**e**) Semilog plots of D^2^ over F_ESA_ for ESA based ablation at different λ_ESA_ with (**b**) λ = 450 nm, (**c**) λ = 500 nm, (**d**) λ = 550 nm, and (**e**) λ = 600 nm. For excitation, different F_pump_ values of 0.2 J/cm² (⁃●⁃), 0.1 J/cm² (▲), and 0.02 J/cm² (▼) were used. F_ESA_ was varied below their respective F_th_ (Table 1), indicated by the vertical dashed line. The values for F_pump_ = 0.2 J/cm² were linearly fit to D^2^ = 0. The diagonal dotted lines highlight the F_th_ fits of λ = 355 nm (---) and the respective λ_ESA_ (---) plotted in (**a**).

It becomes evident from Table 1 that F_th_ of the ESA beams is about one order of magnitude larger than the pump beam. The pump beam with λ_pump_ = 355 nm is absorbed fundamentally by single photon absorption, indicated by the linear increase in D^2^ (at the semilog scale in Figure 3a) and the transmittance in Figure 2a (E_pump_ > E_BG_) [38,39]. In contrast, the ESA beams require either populated defect levels that absorb the laser wavelengths and promote electrons to CB [35], or a multi photon process (E_ESA_ < E_BG_) [7]. However, such nonlinear processes would cause an exponential deviation from the linear trend [38] and is therefore unlikely. Thus, the ESA at defect levels is expected as the primary absorption mechanism. The steeper slope angle of the pump beam indicates a larger beam diameter 2ω_f_ = 28 µm [40]. It is comparable or larger than the ESA beams (Table 1) and therefore it provides ideal conditions for optimal utilization of the pulse energy for the ESA process.

**Figure 4:**
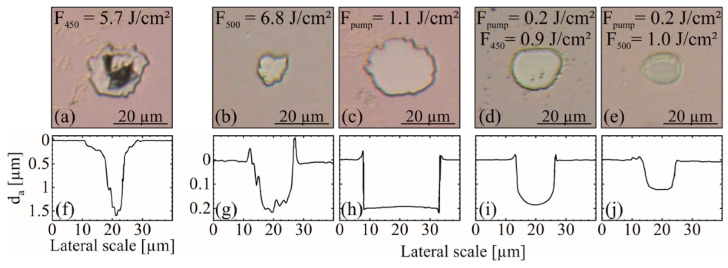
Microscopic images of single-beam ablation spots with (**a**) F_450_ = 5.7 J/cm², (**b**) F_500_ = 6.8 J/cm², and (**c**) F_pump_ = 1.1 J/cm², as well as ESA-based dual-beam ablation spots at (**d**) Δt = 5 ns with F_pump_ = 0.2 J/cm² + F_450_ = 0.9 J/cm² and (**e**) Δt = 10 ns with F_pump_ = 0.2 J/cm² + F_500_ = 1.0 J/cm². (**f**–**j**) White light interferometric depth profiles of spots (**a**–**e**).

The behavior at λ_ESA_ = 450 nm deviates slightly from the other wavelengths, where F_th_ is smaller and 2ω_f_ is larger. This suggests a partially different excitation process due to the high E_P_ closest to E_BG_. This might be explained by the excitation of VB-electrons directly to defect levels close to CB. Moreover, defect levels could be able to absorb the beam and excite electrons directly to CB (Figure 2c), which would lower F_th_ [32,35]. As the defect concentration is expected to be highest at the ZnO–fused silica interface, most of the energy would be absorbed here. At sufficient F_ESA_, the abrupt absorption at this interface can cause an explosion-like ablation of the whole layer without the necessity to heat the entire ZnO inside the laser spot. For single-beam ablation of the ESA-beams, the resulting spots are mostly inhomogeneous at low F_ESA_ (Figure 4b,g) and the further increase of F_ESA_ leads to substrate damage (Figure 4a,f). Here, an inhomogeneous ablation with ablation depths d_a_ of several microns were observed in the dark areas of the ablation spot. The intense absorption at the fused silica interface exceeds the substrate ablation threshold and limits the F_ESA_-range significantly. Thus, for the F_th_ calculations, F_ESA_ was decreased and the spots at higher F_ESA_ exceeding the damage threshold of the fused silica substrate were excluded from analysis, as the measured D^2^ are highly inconsistent and do not represent the ablation characteristic of the ZnO-layer.

### 3.3. Dual-Beam Ablation

#### 3.3.1. Influence of the ESA Beam Fluence

At first, F_ESA_ was varied (Figure 3b–e) and the values for optimum ablation conditions were set to a small positive temporal delay of Δt = 5 ns and F_pump_ = 0.2 J/cm². In the case of an ablation process solely based on ESA, the pump beam induces the transient absorption of the spatially and temporally superimposed ESA beam (Figure 1). This requires to prevent surface modification of the individual beams by ablation [8]. Therefore, all measurements were performed using laser peak fluences below their respective F_th_ (Table 1). This is indicated in Figure 3b–e where all values are below the vertical dashed lines, originating at F_th_ of the respective ESA-beams. Additionally, to compare the dual-beam process with single-beam ablation, the diagonal dashed lines show the linear fits of the F_th_ calculations, determined by the fits of E_th_ in Figure 3a.

Decreasing F_ESA_ results in a decreased D^2^ as less additional energy is available to further excite the already excited electrons. This limits the ablation to an area closer to the beam center, where the intensity of the Gaussian beam profile is still sufficient for ablation. Here, the utilizable fluence range for ESA-based ablation extends about one order of magnitude down to F_ESA_ ≈ 0.3 J/cm² for λ = 450 nm. Slightly below these values, the ablation spots are characterized by their non-circular shape. By further decreasing F_ESA_, inhomogeneously distributed substructures were observed within the irradiated area. These surface modifications at low laser fluences are caused by locally enhanced carrier densities that are generated by either an inhomogeneous laser beam profile or locally enhanced ablation at dust, scratches, or crystal defects [41]. It has to be noted that the analysis only includes ablated structures that resulted in distinct and well pronounced ablation spots, excluding the aforementioned value-ranges.

The comparison of the different ESA wavelengths reveals a similar behavior of the beams with the highest photon energy at λ = 450 and 500 nm showing highly increased D^2^ when compared to λ = 550 and 600 nm at similar fluences (Figure 3b–e). Higher E_P_ allow an increased number of populated defect levels to absorb these ESA beams and to populate the CB by an ESA (Figure 2c). It has to be noted that a single photon absorption of VB-electrons directly to the CB is not possible at λ_ESA_ with E_P_ < E_BG_. In addition, a multiphoton absorption is negligible at the low applied fluences. Furthermore, a transition to another defect level is unlikely due to the specific required wavelength. Thus, besides the intra-band transition of CB-electrons, a transition from defect levels close to the VB into the CB is the most likely absorption mechanism. The existence of a considerable amount of these defect levels is confirmed by the characteristic fluorescence (Figure 2a), where the highest number of emitted photons is attributed to these defect level transitions [42,43,44].

Thus, with the highest E_P,_ using λ_ESA_ = 450 nm is optimal to generate the biggest ablation spots at the lowest F_ESA_ (Figure 3b). Here, at F_pump_ = 0.2 J/cm² (red markers), even less additional F_ESA_ was necessary than the single pump beam requires for ablation. This is indicated by values above the purple line (linear F_th_-fit of Figure 3a).

To analyze the effect of F_pump_ on the ESA-based ablation, Δt = 5 ns at the identical values of F_ESA_ for each wavelength were used. As seen in Figure 3d,e, below F_pump_ = 0.2 J/cm² no distinct ablation spots can be observed for λ = 550 and 600 nm. The utilizable F_ESA_ range reduces drastically with decreasing F_pump_. Without a sufficient excited electron concentration, the ESA-based ablation process stops abruptly. Only for λ = 450 nm at F_pump_ = 0.02 J/cm² distinct ablation spots can be observed (Figure 3b,c).

#### 3.3.2. Ablation Efficiency

When compared to single-beam ablation, the ESA-based process allows to decrease the amount of laser energy required for ablation (Table 1). This improvement was evaluated based on the method of Liu [25]. For this purpose, D^2^ of the ESA-based ablation spots in Figure 3b–e (for F_pump_ = 0.2 J/cm² at Δt = 5 ns) was plotted in a semilog plot as a function of F_ESA_. The linear extrapolation allows to calculate the effective ESA-beam diameter 2ω_f_^eff^ (slope of the fit) and the effective ESA-beam threshold peak fluence F_th_^eff^ (fit intersect at D^2^ = 0) (Table 2).

Here, the values of 2ω_f_^eff^ for λ = 450 and 500 nm are close to their respective 2ω_f_ of single-beam ablation. Consequently, almost the entire area of the fluence profile exceeds the ablation threshold and contributes to the ESA-based ablation. This suggests an effective utilization of the pulse energy. For λ = 550 and 600 nm, the values deviate significantly by a factor of two, which becomes evident by the decreased slope of the fits in Figure 3d,e.

Similarly to F_th_ that defines the minimum required fluence for single-beam ablation, F_th_^eff^ defines the minimum F_ESA_ at given F_pump_ that is required for ESA-based dual-beam ablation. These values decreased by a factor of 5–10, but are still proportional to F_th_. With the highest E_P_, λ_ESA_ = 450 nm can utilize the energy, induced by the pump, most optimally. Here, F_th_^eff^ = 0.26 J/cm is even below F_th_ = 0.49 J/cm² of the single pump beam, indicated by values above the purple line, as described earlier (Figure 3b).

To evaluate the improvements of the ESA-based process, compared to single-beam ablation, fluence efficiency

(3) $$F_{E}=\frac{F_{\mathrm{th}}^{\mathrm{eff}}\;+\;F_{\mathrm{pump}}}{F_{\mathrm{th}}^{\mathrm{pump}}}$$

and total fluence efficiency as the sum of the normalized single-beam fluences

(4) $$F_{\mathrm{ET}}=\frac{F_{\mathrm{th}}^{\mathrm{eff}}\;}{F_{\mathrm{th}}^{\mathrm{ESA}}}\;+\;\frac{F_{\mathrm{pump}}\;}{F_{\mathrm{th}}^{\mathrm{pump}}}$$

were defined (Table 2). For F_E_-values below one, the combined fluences of the ESA- and the pump-beam are still below F_th_^pump^ for single-beam ablation (F_E_ = 1, if F_th_^eff^ + F_pump_ = F_th_^pump^). As already mentioned, only λ = 450 nm at Δt = 5 ns and F_pump_ = 0.2 J/cm² reached F_E_ = 0.86. Similarly, replacing the single-beam threshold fluence in Equation (3) to F_th_ of the ESA-beams, allows the comparison of the efficiency improvement with the ESA-beams. In order to exclude an ordinary accumulation effect of the pump and ESA beam, F_ET_ was defined. For F_ET_ < 1, an additional induced transient absorption must be considered as part of the ablation process (Table 2).

#### 3.3.3. Ablation Quality

Figure 4a,b show selected single-beam ablation spots for the utilized ESA wavelengths. At high F_ESA_, the absorption of defect levels at the fused silica–ZnO interface leads to substrate damage (Figure 4a,f). At F_ESA_ close to F_th_, the ablation spots become increasingly inhomogeneous (Figure 4b). As described earlier, the further decrease of F_ESA_ results in only partially interconnected spots inside the beam. In comparison, the pump beam hits the ZnO–air interface first (Figure 1b) and the fundamental absorption leads to an almost circular and homogeneous single-beam ablation of the entire ZnO layer (Figure 4c). When reaching the ZnO–substrate interface, the pump beam is already highly attenuated preventing abrupt heating and damaging of the substrate (Figure 4h). The maximum d_a_ observed in the depth profiles (Figure 4g–j) are in good agreement with the calculated layer thickness d_L_ = 203 nm.

Compared to single-beam ablation, the spot diameters are decreased by the ESA-based ablation process, indicated by decreased values of 2ω_f_^eff^ when compared to 2ω_f_ (Table 1 and Table 2). The ablation area is restricted by the superposition of both pulses. This limits the ablation to the mostly uniform high-intensive center of the pulses and results in an enhanced ablation quality with circular ablation spots. In comparison to single pump beam ablation, the absorbed energy and induced heat at the ZnO–air interface is reduced and homogeneously distributed throughout the entire ZnO-layer. This results in a reduced melt formation at the edges protruding above the layer surface (Figure 4g–j), as well as decreasing the surface contamination by resolidified ZnO-droplets around the ablation spots (Figure 4d,e). By increasing Δt, these effects are reduced even further, as the heat dissipates and an ESA at defect levels dominates the ablation process (Figure 4i,j).

The optimization of the parameters allows the complete removal of the ZnO layer without damaging the fused silica substrate (Figure 4d,i) and a precise control of d_a_ (Figure 4e,j). The reduced F_ESA_ prevents the fused silica–ZnO interface from abruptly heating to the point of substrate damage (Figure 4a,f). As the fundamentally absorbed pump beam is focused on the ZnO–air surface, the initial abrupt heating together with a high concentration of excited carriers diffuse gradually and homogeneously through the ZnO-layer (d_p_ = 62 nm). Optimal ablation conditions were found at Δt ≈ 5 ns. The ZnO layer is already excited by the pump beam, in particular at the ZnO–substrate interface, where the defects are located primarily. It thus requires only a low excess of additionally absorbed ESA photons and energy to ablate the ZnO layer from the fused silica substrate.

#### 3.3.4. Influence of Temporal Pulse Delay

**Figure 5:**
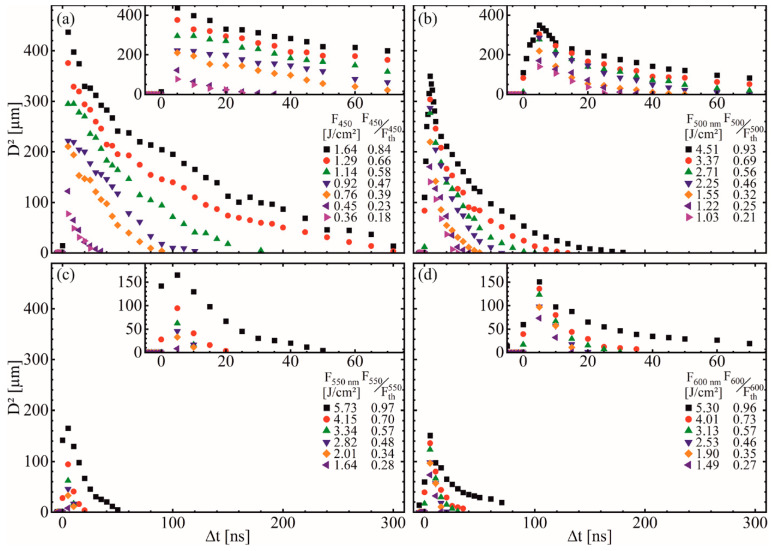
D^2^ plotted versus Δt to determine the optimal delay between the pulses. For excitation, F_pump_ = 0.2 J/cm² was used at (**a**) λ_ESA_ = 450 nm, (**b**) λ_ESA_ = 500 nm, (**c**) λ_ESA_ = 550 nm, (**d**) λ_ESA_ = 600 nm. F_ESA_ was varied in the identical increments as for Figure 3b (color coded with F_ESA_ and the F_ESA_/F_th_-ratio). In the top-right corners, the section of short Δt is extended.

To determine the importance of defect levels compared to absorption solely based on intra-band transition, the temporal delay between the pulses was varied for different F_ESA_ at the fixed optimum of F_pump_ = 0.2 J/cm (Figure 5). At the highest F_ESA_, for λ = 450 and 500 nm (Figure 5a,b), delays above 300 ns still resulted in a measurable ablation. Compared to λ = 550 and 600 nm (Figure 5c,d), this delay is much larger with Δt mostly below 50 ns. As the fluorescent lifetime of ZnO band gap electrons is well below 10 ns [42,44], an ablation at these high Δt cannot be explained solely by intra-band transition-based absorption. Thus, the defect levels with their increased fluorescent lifetime serve as the main driving force [35]. First, the pump beam promotes electrons into CB (Figure 1a). From there, besides relaxation back to the VB, electrons transfer rapidly to deep defect levels, which are suitable as absorption centers for the subsequent ESA beam (Figure 2c). The decrease of D^2^ and the Δt-range is almost proportional to F_ESA_. Only F_ESA_-values close to their respective F_th_ deviate from this trend. Especially for λ = 550 and 600 nm, these values increase overproportional at the highest F_ESA_, where the ESA-beams can almost ablate the ZnO solely by single-beam ablation.

Figure 5c,d reveals deviations from this trend, especially for λ = 550 and 600 nm, where the values increase overproportionately at the highest values of F_ESA_. These fluence values are close the respective ablation thresholds, which almost allow ablation of the ZnO layer solely by the ESA beam.

At low temporal delays (Δt < 10 ns), the impact of λ_ESA_ is less pronounced (Figure 5). Here, the effect of an intra-band transition and sample heating dominates as the beams still overlap. For λ = 550 and 600 nm, an increasing Δt leads to a rapidly decreasing D^2^ because the defect level absorption emerging as the dominant process is only utilized effectively at λ = 450 and 500 nm. As shown in Figure 1c, at Δt = +5 and −5 ns, the temporal overlap is similar, but the resulting D^2^ differ significantly, as prior excitation of electrons by the pump beam is required for ESA.

Exemplary, for λ = 500 nm at F_ESA_ = 4.51 J/cm², Δt was varied in one ns-steps between Δt = −5 to +10 ns to analyze the area of temporal beam overlap in more detail (Figure 5b). At the optimum conditions around Δt ≈ 5 ns, the highest number of prior excited electrons is available for an ESA (Figure 1c). At Δt = 0 ns, where both beams overlap optimally, the excited electrons are only partially used for the ESA, as parts of the radiation excite carriers after the highest intensity of the ESA beam reaches the sample. At negative temporal delays and further decreasing beam overlap, only minor ablation is observed for F_ESA_ close to F_th_. Here, the number of excited electrons is insufficient to reach the required ablation threshold. Increasing Δt above the optimum also leads to a decreasing D^2^-values. However, the rate is much lower than for negative delays.

At Δt < 50 ns, the heat generated by the pump excitation must be considered, as it effects the ablation process considerably, particularly close to the temporal beam overlap (Figure 1c). Here, increased sample temperatures act as an offset for the ESA, as less additionally absorbed energy is required for ablation. Thus, a wider area of the ESA beam surpasses F_th_, resulting in increased D^2^. F_pump_ = 0.2 J/cm², well below F_th_ = 0.49 J/cm² was used to reduce sample heating.

For polycrystalline ZnO-layers, thermal conductivities k_th_ < 10 W/m·K are found in literature, which is well below k_th_ ~ 30–100 W/m·K for epitaxially grown or bulk ZnO [45,46,47,48,49]. The thermal diffusion length is determined by µ = 2(α_th_·Δt)^0.5^ with α_th_ = k_th_/c_V_ where c_V_ is the volumetric heat capacity with about 2.4–2.8·10^6^ J/m³·K [47,49]. With d_p_ = 62 nm, most of the heat is created by the pump beam at the ZnO–air interface. It takes about 5 ns to reach the ZnO–fused silica interface, where the highest defect concentration and thus, the strongest ESA is expected. This is in good agreement with the ablation maximum measured at a delay of about 5 ns.

Furthermore, the rapid D^2^-decrease in the delay range 20–50 ns (Figure 5) can be attributed to heat dissipation and correlates with literature values [45,50]. As 2ω_f_ is about 100 times larger than d_L_ and µ for the time scale in consideration, one-dimensional heat conduction can be assumed [47,51,52]. Here, the heat should be mostly dissipated. Consequently, the defect level absorption is now the dominant factor for ESA, and it is no longer superimposed by pump beam heating. At higher Δt, the ablation slowly decreases due to the finite fluorescent lifetime of the defect level electrons that slowly lowers the available excited electron density for the ESA [43,53].

## 4. Conclusions

In the present study, we investigated a dual-beam laser process based on ESA by applying several wavelengths (λ_pump_ = 355 nm, λ_ESA_ = 450, 500, 550, 600 nm) at various F_pump_ and F_ESA_. The ESA-beams with the highest photon energies produced the largest ablation spots at the lowest beam fluences. A small temporal delay of +5 ns is optimal for the dual-beam ablation process, as it utilizes a combination of heat and excited carriers for increased absorption. Longer temporal delays result in heat dissipation and quickly lower the ablation diameters. Now, the ESA beam absorption is determined solely by the excited carrier concentration. The quality of the ablation spots was improved by reducing the debris and melt formation and the required laser energy was decreased. Furthermore, a model for the evaluation of the ESA-based efficiency improvement was developed. Based on these findings, future investigations on a combination of STED and ESA with regards to STED direct writing processes were advanced.

## Figures and Tables

**Table 1:** Optical parameters (T_E_, n, α, d_p_) at the utilized λ calculated from the transmittance data by the method of Swanepoel (Figure 2a) [26], as well as ablation parameters for single-beam ablation (E_th_, 2ω_f_, F_th_) determined by the method of Liu (Figure 3a) [25].

λ [nm]	355	450	500	550	600
T_E_	0.04	0.82	0.82	0.83	0.83
n	-	2.07	2.05	2.04	2.03
α [1/cm]	1.6 × 10^5^	9.7 × 10^3^	9.5 × 10^3^	9.4 × 10^3^	9.2 × 10^3^
d_p_ [nm]	62	1027	1047	1065	1088
2ω_f_ [µm]	28.4	28.5	20.2	23.6	19.8
E_th_ [µJ]	1.6	6.2	7.8	12.9	8.5
F_th_ [J/cm²]	0.49	1.96	4.86	5.90	5.51

**Table 2:** Calculation of dual-beam ablation parameters (2ω_f_^eff,^ F_th_^eff^) based on the method of Liu [25] and dual-beam efficiency parameters (F_E_, F_ET_) determined from Equations (3) and (4).

λ [nm]	450	500	550	600
2ω_f_^eff^ [µm]	21.4	20.1	12.8	13
F_th_^eff^ [J/cm²]	0.26	0.83	0.90	0.55
F_E_	0.86	2.03	2.16	1.46
F_ET_	0.39	0.50	0.48	0.43

## References

[1] Hell S.W., Wichmann J. (1994). Breaking the diffraction resolution limit by stimulated emission: Stimulated-emission-depletion fluorescence microscopy. Opt. Lett..

[2] Klar T.A., Hell S.W. (1999). Subdiffraction resolution in far-field fluorescence microscopy. Opt. Lett..

[3] Rittweger E., Han K.Y., Irvine S.E., Eggeling C., Hell S.W. (2009). STED microscopy reveals crystal colour centres with nanometric resolution. Nat. Photon..

[4] Gottfert F., Wurm C.A., Mueller V., Berning S., Cordes V.C., Honigmann A., Hell S.W. (2013). Coaligned dual-channel STED nanoscopy and molecular diffusion analysis at 20 nm resolution. Biophys. J..

[5] Malinauskas M., Farsari M., Piskarskas A., Juodkazis S. (2013). Ultrafast laser nanostructuring of photopolymers: A decade of advances. Phys. Rep..

[6] Frolich A., Fischer J., Zebrowski T., Busch K., Wegener M. (2013). Titania woodpiles with complete three-dimensional photonic bandgaps in the visible. Adv. Mater..

[7] Sugioka K., Meunier M., Piqué A. (2010). Laser Precision Microfabrication.

[8] Engel S., Wenisch C., Gräf S., Müller F. (2020). Sub-diffraction direct laser writing by a combination of STED and ESA. Laser-Based Micro-and Nanoprocessing XIV.

[9] Wenisch C., Engel S., Gräf S., Müller F.A. (2020). Fundamentals of a new sub-diffraction direct laser writing method by a combination of stimulated emission depletion and excited state absorption. J. Laser Micro Nanoeng..

[10] Théberge F., Chin S. (2005). Enhanced ablation of silica by the superposition of femtosecond and nanosecond laser pulses. Appl. Phys. A.

[11] Zhang J., Sugioka K., Takahashi T., Toyoda K., Midorikawa K. (2000). Dual-beam ablation of fused silica by multiwavelength excitation process using KrF excimer and F2 lasers. Appl. Phys. A.

[12] Obata K., Sugioka K., Akane T., Midorikawa K., Aoki N., Toyoda K. (2002). Efficient refractive-index modification of fused silica by a resonance-photoionization-like process using F 2 and KrF excimer lasers. Opt. Lett..

[13] Sugioka K., Wada S., Tashiro H., Toyoda K. (1997). Ablation of wide band-gap materials by multi-wavelength irradiation using a VUV Raman laser. Appl. Surf. Sci..

[14] Zhang J., Sugioka K., Wada S., Tashiro H., Toyoda K. (1997). Direct photoetching of single crystal SiC by VUV-266 nm multiwavelength laser ablation. Appl. Phys. A.

[15] Zhang J., Sugioka K., Wada S., Tashiro H., Toyoda K., Midorikawa K. (1998). Precise microfabrication of wide band gap semiconductors (SiC and GaN) by VUV–UV multiwavelength laser ablation. Appl. Surf. Sci..

[16] Akane T., Sugioka K., Hammura K., Aoyagi Y., Midorikawa K., Obata K., Toyoda K., Nomura S. (2001). GaN ablation etching by simultaneous irradiation with F 2 laser and KrF excimer laser. J. Vac. Sci. Tech. B Microelectron. Nanometer Struct. Proces. Meas. Phenom..

[17] Obata K., Sugioka K., Midorikawa K., Inamura T., Takai H. (2006). Deep etching of epitaxial gallium nitride film by multiwavelength excitation process using F 2 and KrF excimer lasers. Appl. Phys. A.

[18] Zoppel S., Zehetner J., Reider G.A. (2007). Two color laser ablation: Enhanced yield, improved machining. Appl. Surf. Sci..

[19] Zoppel S., Merz R., Zehetner J., Reider G.A. (2005). Enhancement of laser ablation yield by two color excitation. Appl. Phys. A.

[20] Djurišić A., Ng A.M.C., Chen X. (2010). ZnO nanostructures for optoelectronics: Material properties and device applications. Prog. Quantum Electron..

[21] Ogata K., Sakurai K., Fujita S., Fujita S., Matsushige K. (2000). Effects of thermal annealing of ZnO layers grown by MBE. J. Cryst. Growth.

[22] Marković S., Simatović I.S., Ahmetović S., Veselinović L., Stojadinović S., Rac V., Škapin S.D., Bogdanović D.B., Častvan I.J., Uskoković D. (2019). Surfactant-assisted microwave processing of ZnO particles: A simple way for designing the surface-to-bulk defect ratio and improving photo (electro) catalytic properties. RSC Adv..

[23] Wang X., Lim G., Zheng H., Ng F., Liu W., Chua S. (2004). Femtosecond pulse laser ablation of sapphire in ambient air. Appl. Surf. Sci..

[24] Rudolph P., Bonse J., Krüger J., Kautek W. (1999). Femtosecond-and nanosecond-pulse laser ablation of bariumalumoborosilicate glass. Appl. Phys. A.

[25] Liu J. (1982). Simple technique for measurements of pulsed Gaussian-beam spot sizes. Opt. Lett..

[26] Swanepoel R. (1983). Determination of the thickness and optical constants of amorphous silicon. J. Phys. E Sci. Instrum..

[27] Sernelius B.E., Berggren K.-F., Jin Z.-C., Hamberg I., Granqvist C.G. (1988). Band-gap tailoring of ZnO by means of heavy Al doping. Phys. Rev. B.

[28] Tauc J., Grigorovici R., Vancu A. (1966). Optical properties and electronic structure of amorphous germanium. Phys. Stat. Solidi B.

[29] Farrag A.A.-G., Balboul M.R. (2017). Nano ZnO thin films synthesis by sol–gel spin coating method as a transparent layer for solar cell applications. J. Sol. Gel Sci. Technol..

[30] Shaaban E., Yahia I., El-Metwally E. (2012). Validity of Swanepoel’s method for calculating the optical constants of thick films. Acta Phys. Pol. Ser. A Gen. Phys..

[31] Xue S., Zu X., Zhou W., Deng H., Xiang X., Zhang L., Deng H. (2008). Effects of post-thermal annealing on the optical constants of ZnO thin film. J. Alloys Compd..

[32] Xu P., Sun Y., Shi C., Xu F., Pan H. (2003). The electronic structure and spectral properties of ZnO and its defects. Nucl. Instrum. Methods Phys. Res. Sec. B Beam Interact. Mater. Atoms.

[33] Wang T., Zheng S., Hao W., Wang C. (2002). Studies on photocatalytic activity and transmittance spectra of TiO2 thin films prepared by rf magnetron sputtering method. Surf. Coat. Technol..

[34] Caglar M., Caglar Y., Ilican S. (2006). The determination of the thickness and optical constants of the ZnO crystalline thin film by using envelope method. J. Optoelectron. Adv. Mater..

[35] Han N.S., Shim H.S., Seo J.H., Kim S.Y., Park S.M., Song J.K. (2010). Defect states of ZnO nanoparticles: Discrimination by time-resolved photoluminescence spectroscopy. J. Appl. Phys..

[36] Lin B., Fu Z., Jia Y. (2001). Green luminescent center in undoped zinc oxide films deposited on silicon substrates. Appl. Phys. Lett..

[37] Johnson J.C., Knutsen K.P., Yan H., Law M., Zhang Y., Yang P., Saykally R.J. (2004). Ultrafast carrier dynamics in single ZnO nanowire and nanoribbon lasers. Nano Lett..

[38] Sugioka K., Midorikawa K. (2013). VUV-UV multiwavelength excitation process for high-quality ablation of fused silica. Damage to VUV, EUV, and X-Ray Optics IV; and EUV and X-Ray Optics: Synergy between Laboratory and Space III.

[39] Mardare D., Tasca M., Delibas M., Rusu G. (2000). On the structural properties and optical transmittance of TiO2 rf sputtered thin films. Appl. Surf. Sci..

[40] Martin S., Hertwig A., Lenzner M., Krüger J., Kautek W. (2003). Spot-size dependence of the ablation threshold in dielectrics for femtosecond laser pulses. Appl. Phys. A.

[41] Bonse J., Baudach S., Krüger J., Kautek W., Lenzner M. (2002). Femtosecond laser ablation of silicon–modification thresholds and morphology. Appl. Phys. A.

[42] Layek A., Manna B., Chowdhury A. (2012). Carrier recombination dynamics through defect states of ZnO nanocrystals: From nanoparticles to nanorods. Chem. Phys. Lett..

[43] Tang A.-H., Mei Z.-X., Hou Y.-N., Du X.-L. (2018). Photodynamics of GaZn–VZn complex defect in Ga-doped ZnO. Chin. Phys. B.

[44] Özgür Ü., Alivov Y.I., Liu C., Teke A., Reshchikov M., Doğan S., Avrutin V., Cho S.-J., Morkoç A.H. (2005). A comprehensive review of ZnO materials and devices. J. Appl. Phys..

[45] Huang Z.X., Tang Z.A., Yu J., Bai S. (2011). Thermal conductivity of nanoscale polycrystalline ZnO thin films. Phys. B Condens. Matter.

[46] Akhir R.M., Abd Wahab Z. (2015). Thermal diffusivity studies of ZnO-CuO at high temperatures. J. Teknologi.

[47] Yamashita Y., Honda K., Yagi T., Jia J., Taketoshi N., Shigesato Y. (2019). Thermal conductivity of hetero-epitaxial ZnO thin films on c-and r-plane sapphire substrates: Thickness and grain size effect. J. Appl. Phys..

[48] Alvarez-Quintana J., Martínez E., Pérez-Tijerina E., Pérez-García S., Rodríguez-Viejo J. (2010). Temperature dependent thermal conductivity of polycrystalline ZnO films. J. Appl. Phys..

[49] Xu Y., Goto M., Kato R., Tanaka Y., Kagawa Y. (2012). Thermal conductivity of ZnO thin film produced by reactive sputtering. J. Appl. Phys..

[50] Nedyalkov N., Koleva M., Nikov R., Atanasov P., Nakajima Y., Takami A., Shibata A., Terakawa M. (2016). Laser nanostructuring of ZnO thin films. Appl. Surf. Sci..

[51] Diez M., Ametowobla M., Graf T. (2017). Time-resolved reflectivity and temperature measurements during laser irradiation of crystalline silicon. J. Laser Micro Nanoeng..

[52] Jiang P., Qian X., Yang R. (2017). Time-domain thermoreflectance (TDTR) measurements of anisotropic thermal conductivity using a variable spot size approach. Rev. Sci. Instrum..

[53] Zhong Y., Djurisic A.B., Hsu Y.F., Wong K.S., Brauer G., Ling C.C., Chan W.K. (2008). Exceptionally long exciton photoluminescence lifetime in ZnO tetrapods. J. Phys. Chem. C.

